# Causal inference between immune cells and glioblastoma: a bidirectional Mendelian randomization study

**DOI:** 10.7150/jca.100519

**Published:** 2025-01-01

**Authors:** Shiqiang Hou, Chunjing Jin, Beitian Shi, Yinan Chen, Ning Lin

**Affiliations:** 1 Department of Neurosurgery, The Affiliated Chuzhou Hospital of Anhui Medical University, The First People's Hospital of Chuzhou, Chuzhou, China.; 2 Laboratory Medicine Center, The Affiliated Chuzhou Hospital of Anhui Medical University, The First People's Hospital of Chuzhou, Chuzhou, China.; 3 Department of Neurosurgery, The First Affiliated Hospital of USTC, Division of Life Sciences and Medicine, University of Science and Technology of China, Hefei, China.

**Keywords:** Mendelian randomization, immune cells, glioblastoma, causal inference, instrumental variables

## Abstract

**Background:** Glioblastoma (GBM) and immunology are closely related, but its mechanism remains unclear. This study aimed to observe the causal inference between GBM and various immune cells by bidirectional Mendelian randomization (MR) analysis.

**Methods:** We used immune cell and GBM data from the GWAS database. A total of 731 immunophenotypes, including four trait types and seven panels. For bidirectional MR analysis, Inverse Variance Weighted and False Discovery Rate (FDR) were both employed. Sensitivity analysis was also performed to make sure the results were reliable.

**Results:** According to FDR, seven immunophenotypes associated with GBM risk: CD33br HLA DR+ AC (FDR = 0.009), CD38 on PB/PC (FDR = 0.046), CD66b on CD66b++ myeloid cell (FDR = 0.019), CD3 on CD39+ resting Treg (FDR = 0.009), HVEM on CM CD8br (FDR = 0.050), CD45 on CD33br HLA DR+ CD14dim (FDR = 0.027), and CD86 on CD62L+ myeloid DC (FDR = 0.048). In reverse MR analysis, GBM was found to be strongly associated with nine immunophenotypes based on FDR: BAFF-R on CD24+ CD27+ (FDR = 0.033), BAFF-R on IgD+ CD38- (FDR = 0.036), BAFF-R on IgD-CD38br (FDR = 0.039), BAFF-R on unsw mem (FDR = 0.048), BAFF-R on CD20- (FDR=0.012), HVEM on EM CD8br (FDR=0.036), CCR2 on myeloid DC (FDR = 0.035), CD45 on CD33-HLA DR+ (FDR = 0.004), and CD34 on HSC (FDR = 0.035).

**Conclusion:** The current study confirmed the causal inference between 16 different immunophenotypes and GBM using genetic tools, providing an important foundation and guide for future immunological research and immunotherapy of GBM.

## 1. Introduction

The brain is now understood to be an organ intimately connected to the immune system, rather than an immune-isolated organ^1^. Microglia are specialized immune cells in the brain that make up approximately 20% of the glial cells. Microglia are equivalent to macrophages in the central nervous system (CNS) and are the first and most important line of immune defense^2^. In addition, various regions of the meninges, perivascular space, choroid plexus, and brain border contain circulating immune cells that patrol and sense the brain remotely^3^-^6^. According to early studies, the immune system can damage neurons by producing chemicals that cause inflammation and cell death^7^. Later, some researchers found that T cells and other immune cells may have a protective effect on the brain^8^. However, the types and interactions of immune cells are very complex, and the specific mechanism of their action on the brain is still unclear.

Glioblastoma (GBM) is the most aggressive form of glioma and has the poorest prognosis^9^. Although the treatment methods have been continuously improved, the problems of GBM heterogeneity and genetic instability of cancer cells have not been solved, which also leads to the unsatisfactory clinical treatment effect of GBM^10^. Immune checkpoint-based therapy is a new method. The combination of PD-1 monoclonal antibody and oncolytic adenovirus can achieve some effect on recurrent GBM, but only for some patients^11^. In addition, many studies have shown that simple immune checkpoint inhibitors have little effect on GBM^12^, ^13^. The main reason for the limited effect of immunotherapy is that the immune microenvironment of GBM is in an immunosuppressive state, containing large numbers of myeloid-derived suppressor cells (MDSC) and tumor-associated macrophages (TAM), and lacking T-cell infiltration^10^, ^14^.

Prior research has demonstrated the critical role immune cells play in the development of several CNS diseases, such as GBM^2^, ^15^. The tumor microenvironment (TME) of GBM is very important for its malignant progression. Myeloid cells are the most prominent and dominant cells in the TME, including microglia, TAM, neutrophils, and MDSC^16^, ^17^. Myeloid cells are involved in the early stages of the immune response due to their many immunological roles, including phagocytosis, microbial clearance, antigen presentation, and the synthesis of inflammatory mediators^18^, ^19^. Myeloid cells can interact with cancer cells and lymphocytes to induce immunosuppression and promote tumor growth^20^, ^21^. It has been shown by flow cytometry that the TME of early GBM contains mainly microglia, whereas the number of macrophages increases significantly in late GBM, which is highly parallel to the destruction of the BBB and the explosive growth of EGFR+ GBM cells^22^. It has also been shown that macrophages can activate STAT3 through receptor-binding ligand and eventually induce GBM to transform into mesenchymal-like cells^23^. In general, the immune crosstalk and interaction of GBM are very complicated, such as GBM-T cells, GBM-neutrophils and GBM-MDSC^23^, ^24^. In these cells, functional crosstalk can be achieved by secreting various cytokines, chemokines, and exosomes^25^-^27^.

Immune cells are closely related to GBM, and immunotherapy has become an important method to treat GBM in the future. Although many scholars have reported the crosstalk and interaction between immune cells and GBM, the communication and regulation mechanism between them is still unknown, especially the genetic correlation research is less^28^. Mendelian randomization (MR), as a new method to study genetic correlation, takes single nucleotide polymorphisms (SNPs) as instrumental variables (IVs)^29^. MR analysis can accurately predict the genetic correlation and avoid the interference of other factors, which has attracted increasing attention from GBM scholars^30^. This study will deeply explore the causal relationship between immune cells and GBM through two-way MR analysis, and fully explore the genetic correlation between GBM and immune cells, which can provide an important theoretical basis for activating immune cells to induce anti-tumor immune response.

## 2. Materials and Methods

### 2.1 Data sources

We collected SNPs for immune cells and GBM as a study topic from the publicly available GWAS database (https://gwas.mrcieu.ac.uk/datasets/). As shown in **Table S1 and 2**, the GWAS ID for GBM is finn-b-C3_GBM_EXALLC (Ncase = 91, Ncontrol = 174006), for a total of 16380303 SNPs. The GWAS IDs for immune cells were GCST 90001391 to GCST 90002121.These datasets contained a total of 731 immunophenotypes, including four trait types (AC, RC, MFI, and MP) and seven panels (B cell, cDC, Maturation stages of T cell, Monocyte, Myeloid cell, TBNK, Treg)^31^.

### 2.2 Study design

The MR analysis based on a bidirectional two-sample design to thoroughly investigate the causal inference between immune cells and GBM in depth. To obtain accurate results, genetic variants as instrumental variables must satisfy three main assumptions: (1) IVs are strongly correlated with exposure factors (correlation assumption), (2) IVs are independent of confounders (independence assumption), and (3) IVs can only play a role on the outcome through exposure factors (exclusionary hypothesis). No further ethical assessment was necessary because the data for this investigation came from sources that were accessible to the public. Nonetheless, the STROBE-MR standards were closely followed in this investigation^32^, ^33^.

### 2.3 Selection of IVs

Genetic variants were selected as IVs when pruning all linkage imbalance SNPs to reduce confounding and pleiotropy. To ensure that the selected SNP had a strong correlation with immune cell phenotype and GBM, the effect size and significance level of the SNP were further examined. P < 1e-5 and F >10 was used as the screening condition for subsequent MR analysis. All data (**Table S3**) with a P-value less than 0.05 were corrected for FDR, and IVW statistics were also used for subsequent analysis. Finally, FDR< 0.05 is considered as significant.

### 2.4 Statistical analysis

This MR analysis was performed in R software (version 4.3.1). MR-Egger, weighted median, inverse variance weighted (IVW), simple mode, and weighted model were used for MR analysis. In the absence of heterogeneity and multiple effects, IVW was the primary outcome and was reported with OR, 95% CI, P, and FDR. If the results of other methods were not statistically significant, the results were also retained if FDR< 0.05 for IVW and the beta values of all methods were positive or negative. Forest plots generated by meta-analysis clearly show positive and negative associations between immune cells and GBM. Sensitivity analyses were performed to assess the robustness of the results of the MR analyses, using MR-Egger to exclude pleiotropy and discarding results when p<0.05. Heterogeneity was assessed with the Cochran Q test, and the fixed effects model was used when p > 0.05. Furthermore, the leave-one-out method was used to validate the sensitivity analysis by excluding the effect of a particular SNP on the results. The stability, repeatability, and variability of the findings in the MR study were displayed using forest plots, scatter plots, and funnel plots.

## 3. Results

### 3.1 Causal effect of immune cells on GBM

**Figure 1:**
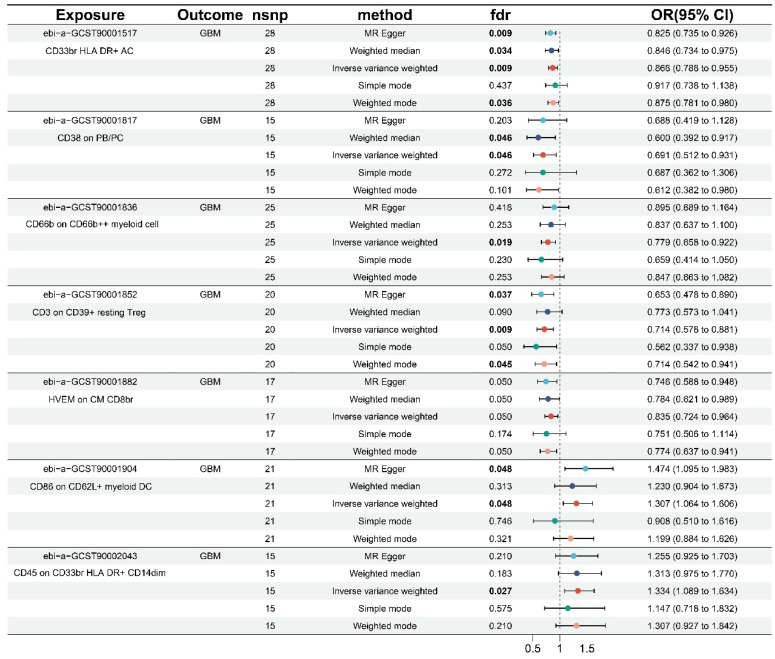
Forest plots of the causal effect of immune cells on GBM by different methods.

As shown in** Table 1** and **Figure S1**, the results of this study were free of heterogeneity and horizontal pleiotropy (all P values > 0.05). Combined with the forest plot we generated (**Figure 1**), it was found that six immunophenotypes were identified when FDR < 0.05. One of them was derived from the AC trait type, which was CD33br HLA DR+ AC (Myeloid cell). Five of them were derived from MFI trait type, including CD38 on PB/PC (B cell), CD66b on CD66b++ myeloid cell (Myeloid cell), CD3 on CD39+ resting Treg (Treg), and CD86 on CD62L+ myeloid DC (cDC), and CD45 on CD33br HLA DR+ CD14dim (Myeloid cell). At FDR = 0.05, an additional immunophenotype derived from the MFI trait type was identified as HVEM on CM CD8br (Maturation stages of T cells).

Five of the seven immunophenotypes were found to be GBM protective: CD33br HLA DR+ AC (OR = 0.868, 95% CI = 0.788-0.955, p = 0.004, FDR = 0.009), CD38 on PB/PC (OR = 0.691, 95% CI = 0.512-0.931, p = 0.015, FDR = 0.046), CD66b on CD66b++ myeloid cell (OR = 0.779, 95% CI = 0.658-0.922, p = 0.004, FDR = 0.019), CD3 on CD39+ resting Treg (OR = 0.714, 95% CI = 0.578-0.881, p = 0.002, FDR = 0.009), and HVEM on CM CD8br (OR = 0.835, 95% CI = 0.724-0.964, p = 0.014, FDR = 0.050). Weighted median (OR = 0.846, 95% CI = 0.734-0.975, p = 0.021, FDR = 0.034), weighted model (OR = 0.875, 95% CI = 0.781-0.980, p = 0.029, FDR = 0.036), and MR-Egger (OR = 0.825, 95% CI = 0.735-0.926, p = 0.003, FDR = 0.009) showed identical results in CD33br HLA DR+ AC. The weighted median results in CD38 on PB/PC (OR = 0.600, 95% CI = 0.392-0.917, p = 0.018, FDR = 0.046) were in agreement with the IVW method. The same results were obtained in the CD3 on CD39+ resting Treg by the weighted model (OR = 0.714, 95% CI = 0.542-0.941, p = 0.027, and FDR = 0.045) and MR-Egger (OR = 0.653, 95% CI = 0.478-0.890, p = 0.015, FDR = 0.037).

The remaining two immunophenotypes, CD45 on CD33br HLA DR+ CD14dim (OR = 1.334, 95% CI = 1.089-1.634, p = 0.005, FDR = 0.027) and CD86 on CD62L+ myeloid DC (OR = 1.307, 95% CI = 1.064-1.606, p = 0.011, FDR = 0.048), were associated with an increased risk of GBM. MR-Egger (OR = 1.474, 95% CI = 1.095-1.983, p = 0.019, FDR = 0.048) for CD86 on CD62L+ myeloid DC was consistent with the IVW results. Additionally, the scatter plots, funnel plots, and leave-one-out analyses in **Figures S2-S4** demonstrated the results and their reliability.

### 3.2 Causal effect of GBM on immune cells

**Figure 2:**
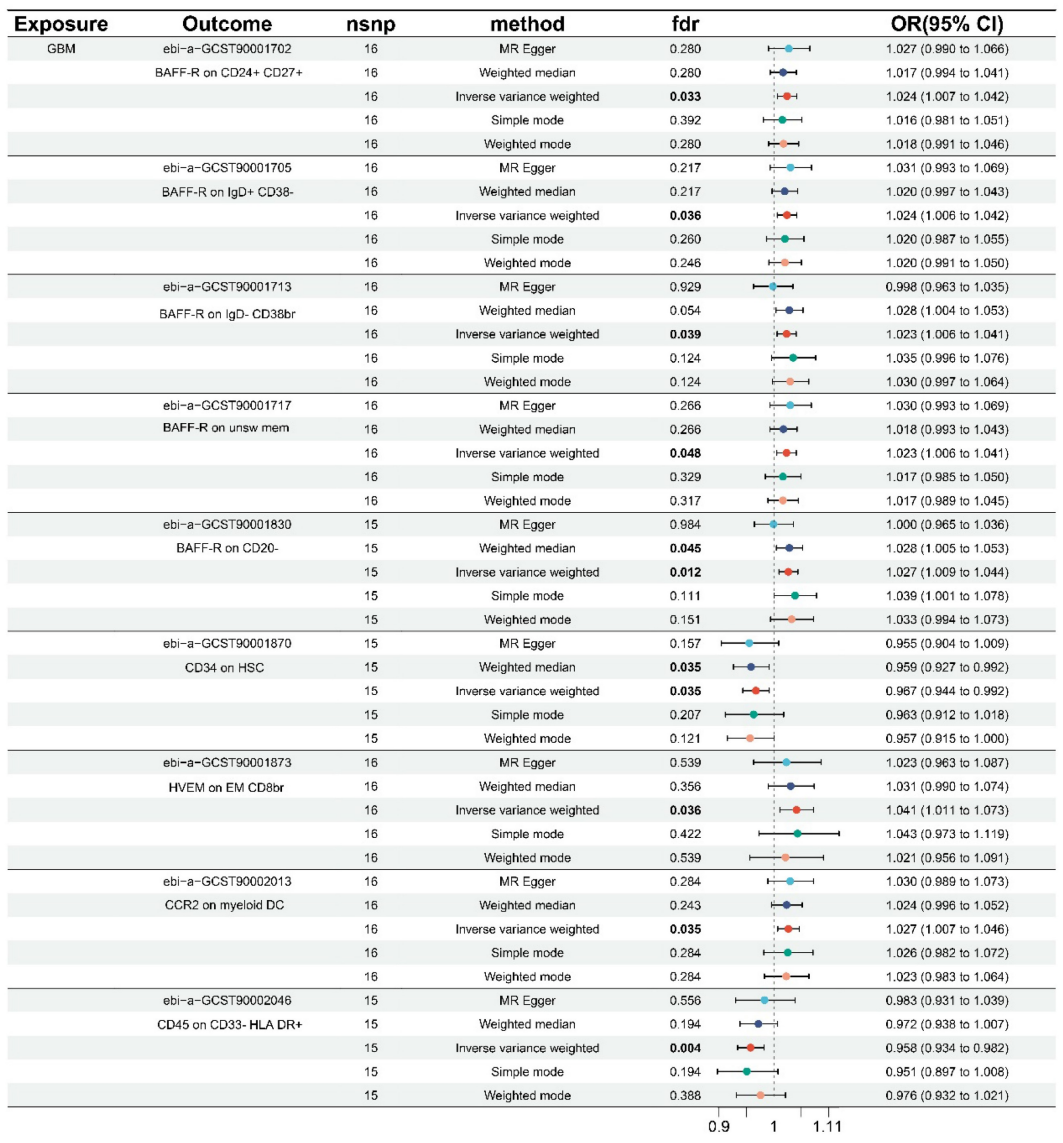
Forest plots of the causal effect of GBM on immune cells by different methods.

Using the same technique, we performed inverse MR analysis to investigate the causal inference between immunophenotype and genetic susceptibility to GBM. Sensitivity analysis revealed no horizontal pleiotropy or heterogeneity, as shown in **Table 2** and **Figure S5**. After FDR correction, the data revealed that a total of 9 immunophenotypes were found when combined with the forest plot (**Figure 2**). All nine immunophenotypes were members of the MFI trait type, with seven immunophenotypes, namely: BAFF-R on CD24+ CD27+ (B cell), BAFF-R on IgD+ CD38- (B cell), BAFF-R on IgD- CD38br (B cell), BAFF-R on unsw mem (B cell), BAFF-R on CD20- (B cell), HVEM on EM CD8br (T cell maturation stages), and CCR2 on myeloid DC (cDC), were negatively correlated with GBM. GBM exhibited a positive correlation with two immunophenotypes: D45 on CD33-HLA DR+ (Myeloid cell) and CD34 on HSC (Myeloid cell).

The IVW results all showed that BAFF-R on CD24+ CD27+ (OR = 1.024, 95% CI = 1.007-1.402, p = 0.007, FDR = 0.033), BAFF-R on IgD+ CD38- (OR = 1.024, 95% CI = 1.006-1.042, p = 0.007, FDR = 0.036), BAFF-R on IgD- CD38br (OR = 1.023, 95% CI = 1.006-1.041, p = 0.008, FDR = 0.039), BAFF-R on unsw mem (OR = 1.023, 95% CI = 1.006-1.041, p = 0.010, FDR = 0.048), BAFF-R on CD20- (OR = 1.027, 95% CI = 1.009-1.044, p = 0.009, FDR=0.012), HVEM on EM CD8br (OR = 1.041, 95% CI = 1.011-1.073, p = 0.007, FDR=0.036), CCR2 on myeloid DC (OR = 1.027, 95% CI = 1.007-1.046, p = 0.007, FDR = 0.035). Consistent results were observed for BAFF-R on CD20-, weighted median (OR = 1.028, 95% CI = 1.005-1.503, p = 0.018, FDR = 0.045).

The IVW results for the remaining two immunophenotypes are as follows: CD45 on CD33-HLA DR+ (OR = 0.958, 95% CI = 0.934-0.982, p = 0.001, FDR = 0.004), and CD34 on HSC (OR = 0.967, 95% CI = 0.944-0.992, p = 0.009, FDR = 0.035). The IVW results were similar with the weighted median in CD34 on HSC (OR = 0.959, 95% CI = 0.927-0.992, p = 0.014, FDR = 0.035). In addition, scatter plots, funnel plots, and leave-one-out analysis in **Figure S6-S8** further demonstrated the robustness of the results.

## 4. Discussion

The concept that the brain is not immune-isolated but depends on the integrity of the immune system has attracted the attention of many scientists since it was proposed in the 21st century^34^. The main evidence has been that cerebrospinal fluid can flush the cranial bone marrow and affect its hematopoietic function, and that the cranial bone marrow is the repository of immune cells before they migrate to the CNS^35^, ^36^. These findings have transformed CNS immune surveillance from microglia to a complex brain immune network with multiple peripheral immune participants^1^. Microglia are the immune cells of the brain that can kill viruses, damaged cells, and various pathogens^2^. In addition, various innate and adaptive immune cells fill the meninges, choroid plexus, and perivascular spaces around the CNS. There is a direct vascular pathway between the dura mater and the skull that can carry immune cells. Under physiological conditions, these vascular channels can transport myeloid cells to the meninges and play the role of sentries, whereas under pathological conditions, myeloid cells located in the meninges infiltrate the brain tissue^37^.

In GBM, the TME is rich in inhibitory immune cells (TAM, MDSC, neutrophils and Treg cells) and deficient in cytotoxic T cells, making GBM a "cold" tumor. Due to the alteration of the blood-brain barrier, more immune-related substances enter the brain tissue, and various immune cells interact with tumor cells in different ways^38^. In short, this microenvironment not only promotes the growth of GBM, but also causes immunosuppressive TME, leading to failure of immunotherapy. TAM can make GBM cells more invasive by reprogramming, and macrophages can also induce GBM cells to transform into mesenchymal-like cells^23^, ^25^. In addition, the progression of GBM may be due not only to drug resistance of cancer cells, but also to immunosuppression induced by MDSC accumulation^22^. Nevertheless, other studies suggest that the primary characteristic of tumor-infiltrating lymphocytes in GBM may not be T-cell depletion^39^. In summary, an increasing number of studies have demonstrated that the TME is critical for the development of GBM. The heterogeneity of cancer cells and TME is an important feature of GBM, such as different epigenetic alterations and different stromal cell types. Although the inheritance of GBM cells is highly heterogeneous and unstable, the inheritance of non-cancerous cells in the TME is relatively stable. Therefore, it is of great importance to elucidate the mechanism of action between GBM and various immune cells in the TME and their genetic relationship.

To better observe the relationship between two diseases or exposure factors and outcomes from a genetic perspective, MR analysis has been widely used in recent years^29^. Some scholars found that three immune cell phenotypes are closely related to GBM through Bayesian MR analysis, but no further data verification was used in the analysis^28^. In this study, we thoroughly discuss the causal relationship between immune cells and GBM through two-way MR and FDR verification, and the results are more rigorous and accurate. The results showed that when immune cells were used as exposure factors, seven immunophenotypes were identified: CD33br HLA DR+ AC (Myeloid cell), CD38 on PB/PC (B cell), CD66b on CD66b++ myeloid cell (Myeloid cell), CD3 on CD39+ resting Treg (Treg), HVEM on CM CD8br (Maturation stages of T cell), CD86 on CD62L+ myeloid DC (cDC), CD45 on CD33br HLA DR+ CD14dim (Myeloid cell). Among them, the first five immunophenotypes are associated with decreased GBM risk, while the last two immunophenotypes are associated with increased GBM risk. When GBM was used as the exposure factor, nine immunophenotypes were identified: BAFF-R on CD24+ CD27+(B cell), BAFF-R on IgD+ CD38- (B cell), BAFF-R on IgD- CD38br (B cell), BAFF-R on unsw mem (B cell), BAFF-R on CD20- (B cell), HVEM on EM CD8br Maturation stages of T cell (Maturation stages of T cell), CCR2 on myeloid DC (cDC), CD34 on HSC (Myeloid cell), CD45 on CD33- HLA DR+ (Myeloid cell).In addition, scatter plot, funnel plot and leave-one-out analysis in this study also show the results visually and prove that the research results are robust.

T cells are lymphocytes that develop into many subsets, including CD4+ and CD8+ T cells, and are critical for the anti-tumor response^40^. Previous studies have shown that the infiltration of CD3+ and CD8+ T cells is the most significant in mesenchymal GBM, while the IDH type is the least significant^41^. CD8+ cells identified in the peripheral blood and tumor of GBM are CD38+ and HLA-DR+^42^. Through a variety of methods and interactions with other cells, CD8+T cells can induce cancer cells to undergo apoptosis. CD 8+T directly kills GBM cancer cells by activating cytotoxic signals through MHC-I, and at the same time, GBM cells can adaptively under-express MHC-I to escape the tumor-killing effect of CD 8+T cells^20^. Numerous immune cell types, including as T, B, and Treg cells, as well as mesenchymal and epithelial cells, express HVEM. Like PD-L1 overexpression, HVEM upregulation in malignancies is an immune escape strategy^43^. The detection results of HVEM in GBM showed that its expression was localized to the surrounding area of necrosis and microvascular hyperplasia area. According to the transcriptome study, HVEM expression is associated with immunological and stromal cell infiltration in the TME, which is crucial for controlling inflammatory and immune responses, especially T-cell activation. In addition, HVEM was negatively correlated with T cell-mediated immune regulation, cytotoxicity regulation, and T cell receptor signal transduction regulation^44^. Therefore, HVEM may be helpful in inhibiting the anti-tumor effect associated with T cells in the GBM microenvironment.

The primary function of Treg cells is to suppress inflammation and the immune response. Studies have shown that Treg infiltration has no significant correlation with overall survival in GBM^45^. Treg cells are critical for immune checkpoint inhibitor resistance, while phase III therapeutic trials of immune checkpoint inhibitors in GBM have not been successful. Encouraging CD4 Treg cells to differentiate into CD4 effector T cells has been shown to attenuate the immunosuppressive response mediated by Treg cells^46^. Treg cells can also prevent CD8+ T cell activation by aggregating in the TME. Additionally, the TME is largely responsible for the immunosuppressive failure of CAR-T in the treatment of solid tumors such as GBM. The accumulation of lactic acid produced by glycolysis of tumor cells will cause CAR-T immunosuppression, and the upregulation of CD39, CD73 and CCR8 is an important mechanism^47^. Reducing Treg cell infiltration by cytokine stimulation is a potentially effective way to realize CAR-T cell therapy in GBM^48^.

Intracranial B cells were found to be derived from cranial hematopoiesis and not from the peripheral circulation^49^. B cell infiltration is present in approximately 40% of GBM patients, and one of the key features of the GBM microenvironment is the suppression of CD8+ T cell activation by invading B cells. B cells express CD20, and studies in animal models have shown that immunotherapy targeting CD20 increases survival^50^. However, in a study of 98 GBM samples, CD20+ cells were observed in only 4 cases^51^. As a receptor for BAFF, BAFF-R is the most important survival-promoting receptor of B cells, and its expression begins as immature B cells develop into transitional B cells^52^. According to a meta-analysis, the expression of BAFF and BAFF-R in gliomas was associated with tumor grade^53^. Vaccine research can greatly benefit from the activation and expansion of CD8+ T cells, which can be facilitated by using BAFF to activate B cells to generate antigen-presenting B cells^54^. Numerous cell types, including bone marrow-derived cells, express CD38. These cells are primarily involved in the processes of cell differentiation and inflammation, and they are critical in the autoimmune inflammatory process^55^. CD38 can suppress CD8+ T cells and is the primary mechanism of resistance to PD-1/PD-L1 blockers^56^. Studies have confirmed that CD38 can regulate microglial activation *in vitro* and *in vivo*, and its absence can inhibit glioma progression^57^. Additionally, a negative correlation between CD38 and glioma cell invasion and apoptosis has been discovered^58^. Therefore, CD38 may be a useful molecular biological marker and a prospective therapeutic target for glioma patients^59^.

As a biomarker of B cells, CD24 can regulate cell differentiation and is also one of the immune checkpoints in GBM^60^. Overexpression of CD24 in GBM is associated with poor overall patient survival, and inactivation of CD24 inhibits GBM cell invasion^61^. B cells are one of several immune cell types that express CD27, a member of the TNF receptor superfamily. CD27 has only one ligand, which is CD70 (CD27L). The CD27-CD70 signaling axis induces T cells to release TNF-α, enhances the activity of cytotoxic CD8+ T cells, and promotes B cell activation and terminal differentiation into plasma cells^62^. It has been found that inducing apoptosis of B cells and T cells through the interaction between CD70 expressed on glioma cells and CD27 expressed on B cells and T cells may be a new method for immune escape of malignant glioma^63^. In CD70+ gliomas, CD68/CD163/HLA-DR+ tumor-associated macrophages were significantly increased, but CD27+ tumor-infiltrating lymphocytes were not significantly increased^64^. Targeted therapy based on CD70 has shown anti-tumor activity and is in clinical trials. To summarize, a growing body of research has verified that stimulation of the CD27-CD70 signaling pathway may serve as an innovative treatment avenue for several cancers, including GBM.

The main function of dendritic cells is to collect, process, and display antigens within the immune system to elicit an immune response from T and B cells^65^. Myeloid DCs are the largest group of dendritic cells that share the same progenitor cells as monocytes and play an important role in antigen processing and control of the initiation of the immune response^66^. CCR2 is a chemokine receptor that can regulate immune cell migration and inflammatory response^67^. A single cell analysis study of GBM showed that immune cells in the TME significantly expressed many genes, including CCR2^68^. In addition, several investigations have demonstrated that the CCR2/CCL2 signaling pathway controls the immunological activity of macrophages and microglia^69^. However, the CCR2/CCL2 signaling pathway has no significant effect on MDSC infiltration in GBM^70^.

CD66b is a biomarker shared by neutrophils and MDSC that can be combined with other biomarkers to identify the phenotype of immune cells^71^. Glioma-derived CD66b+ cells have been shown to specifically express the neutrophil marker gene^72^. In addition, the expression of CD66b+ neutrophils in the perivascular region are associated with GBM prognosis^73^. Dendritic cells, monocytes, and B cells all produce CD86, and a high expression of this protein is associated with poor survival in GBM^74^. In the study of the interaction between CD34+ HSC and GBM cells, it was observed that HSC could exchange fluorescent labels with GBM^75^. Under the condition of hypoxia or glucose deficiency, glioma stem cells can express CD31 and CD34 and participate in angiogenesis^76^. Additionally, studies of GBM bleeding revealed increased expression of CD34, CD105, and angiogenic factors, suggesting a potential role for hypoxia-induced angiogenesis and increased vascular density in GBM bleeding^77^. As a phosphatase on the surface of leukocytes, CD45 can regulate the activity of immune cells^28^. A mouse model study showed that CD45+ macrophages accumulated in advanced GBM, but no similar results were found in contralateral normal brain tissue^22^. Research on immunological markers suggests that HLA DR and CD14 dim may be associated with phagocytosis and the inflammatory response, while CD33br may be associated with anti-inflammatory response, modulation of cell activity, and antigen delivery^78^. In addition, certain studies have verified that MDSC express the myeloid marker CD33 but do not carry the mature myeloid marker HLA-DR^79^.

The relationship between GBM and immune cells, and between immune cells themselves, is complex. Bone marrow cells can transmit immunosuppressive signals from cancer cells to T cells, thereby inhibiting the anti-tumor function of T cells^80^. MDSC can lead to T-cell depletion and transform B cells into regulatory B cells in various ways^50^, ^81^. Genetic alterations in GBM cells have been shown to regulate the biological activities of tumor-associated microglia and macrophages^82^. Many studies have also shown that GBM-derived factors also play an important role in the biological regulation of immune cells^21^. With the deepening understanding of various brain immune cells, immunotherapy targeting GBM-TME has become an important research direction in the treatment of GBM, as the crosstalk between tumor cells and immune cells will affect immunotherapy^83^. By preventing the recruitment or polarization of TAM or MDSC, some success has been achieved in reducing immunosuppression in the solid tumor TME^84^. However, the brain is still a unique area in immunology, and there is still a long way to go in future research on immunotherapy.

Unlike previous studies that focused on a single immunophenotype, this study found that GBM was closely related to genetic variation in 16 immunophenotypes through bidirectional MR and FDR verification. The results of this study and previous studies can not only prove each other, but also complement each other. The results of this study confirmed that TME is the core participant in GBM, and the interaction between immune cells and tumor cells contained in TME plays an important role in promoting the development of GBM. At the same time, this study provides a new perspective to further elucidate the specific biological pathway and molecular mechanism of the causal relationship between immunophenotype and GBM, and also provides a new basis for future immunotherapy and TME-related research. Although the results of this study are based on strict conditions such as sensitivity analysis and horizontal pleiotropic testing, they also have the limitations and shortcomings that are widely recognized in MR analysis^85^. For example, patient information and clinical parameters are not detailed enough for in-depth stratified analysis. In addition, although 731 immune cell phenotypes were included, the source of GBM data was relatively single, limiting the accuracy and universal applicability of the results. In the future, MR analysis based on large samples and basic experimental research will be used to further explore the relationship between GBM and immunophenotype.

## 5. Conclusion

In conclusion, we demonstrated the causal inference between GBM and 16 different immunophenotypes through comprehensive and rigorous bidirectional MR analysis, and further confirmed the complex crosstalk mode between GBM and immune cells. The results of this study provide a new direction and theoretical basis for immune checkpoint inhibitors and TME-based CAR-T therapy.

## Supplementary Material

Supplementary figures and tables 1-2.

Supplementary table 3.

## Figures and Tables

**Table 1:** Causal effect of immunophenotypes genetics IVs on GBM

Exposure	GWAS ID	Outcome	Method	Nsnp	P	FDR	OR (95%CI)	P for heterogeneity	P for pleiotropy
CD33br HLA DR+ AC	ebi-a-GCST90001517	GBM	IVW	28	0.004	0.009	0.868(0.788-0.955)	0.577	0.135
CD38 on PB/PC	ebi-a-GCST90001817	GBM	IVW	15	0.015	0.046	0.691(0.512-0.931)	0.629	0.982
CD66b on CD66b++ myeloid cell	ebi-a-GCST90001836	GBM	IVW	25	0.004	0.019	0.779(0.658-0.922)	0.790	0.188
CD3 on CD39+ resting Treg	ebi-a-GCST90001852	GBM	IVW	20	0.002	0.009	0.714(0.578-0.881)	0.224	0.448
HVEM on CM CD8br	ebi-a-GCST90001882	GBM	IVW	17	0.014	0.050	0.835(0.724-0.964)	0.540	0.266
CD86 on CD62L+ myeloid DC	ebi-a-GCST90001904	GBM	IVW	21	0.011	0.048	1.307(1.064-1.606)	0.553	0.285
CD45 on CD33br HLA DR+ CD14dim	ebi-a-GCST90002043	GBM	IVW	15	0.005	0.027	1.334(1.089-1.634)	0.519	0.611

**Table 2:** Causal effect of GBM genetics IVs on immunophenotypes

Exposure	Outcome	GWAS ID	Method	Nsnp	P	FDR	OR (95%CI)	P for heterogeneity	P for pleiotropy
GBM	BAFF-R on CD24+ CD27+	ebi-a-GCST90001702	IVW	16	0.007	0.033	1.024(1.007-1.042)	0.798	0.854
GBM	BAFF-R on IgD+ CD38-	ebi-a-GCST90001705	IVW	16	0.007	0.036	1.024(1.006-1.042)	0.892	0.705
GBM	BAFF-R on IgD- CD38br	ebi-a-GCST90001713	IVW	16	0.008	0.039	1.023(1.006-1.041)	0.927	0.149
GBM	BAFF-R on unsw mem	ebi-a-GCST90001717	IVW	16	0.010	0.048	1.023(1.006-1.041)	0.845	0.688
GBM	BAFF-R on CD20-	ebi-a-GCST90001830	IVW	15	0.009	0.012	1.027(1.009-1.044)	0.819	0.121
GBM	CD34 on HSC	ebi-a-GCST90001870	IVW	15	0.009	0.035	0.967(0.944-0.992)	0.637	0.623
GBM	HVEM on EM CD8br	ebi-a-GCST90001873	IVW	16	0.007	0.036	1.041(1.011-1.073)	0.308	0.517
GBM	CCR2 on myeloid DC	ebi-a-GCST90002013	IVW	16	0.007	0.035	1.027(1.007-1.046)	0.552	0.860
GBM	CD45 on CD33- HLA DR+	ebi-a-GCST90002046	IVW	15	0.001	0.004	0.958(0.934-0.982)	0.777	0.313
