# Supplementary material for: Causal inference between immune cells and glioblastoma: a bidirectional Mendelian randomization study
Source: J Cancer. 2025 Jan 1;16(1):171–81. doi: 10.7150/jca.100519 (PMC11660118; doi:10.7150/jca.100519)
Supplement: Supplementary file 1 — Supplementary figures and tables 1-2. [file jcav16p0171s1.pdf]

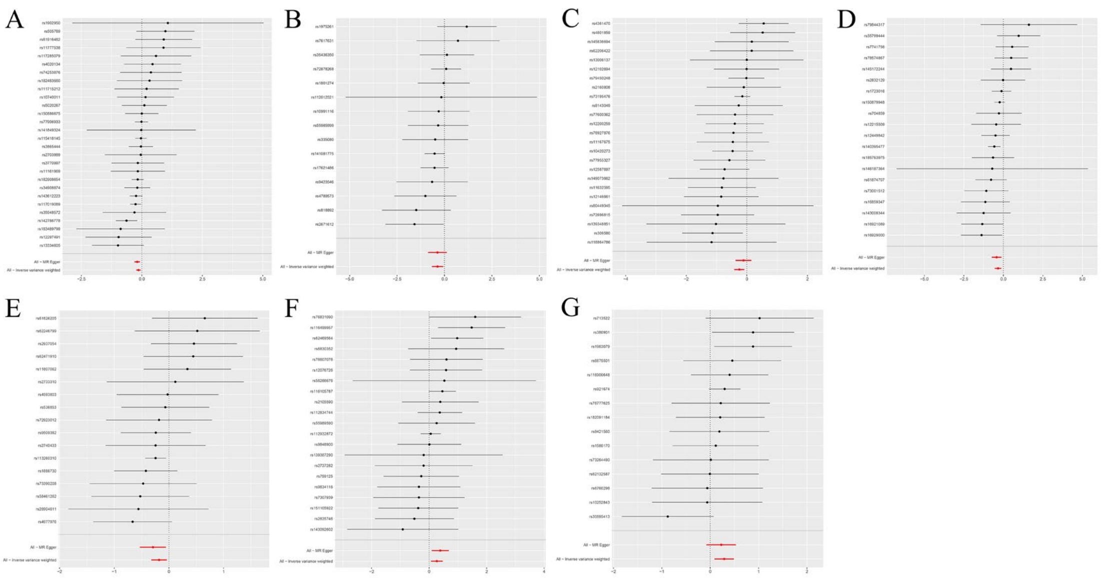

Figure S1. MR effect size for the causal effect of immunophenotypes SNPs on the risk of GBM. (A) ebi-a-GCST90001517, (B) ebi-a-GCST90001817, (C) ebi-a-GCST90001836, (D) ebi-a-GCST90001852, (E) ebi-a-GCST90001882, (F) ebi-a-GCST90001904, (G) ebi-a-GCST90002043.

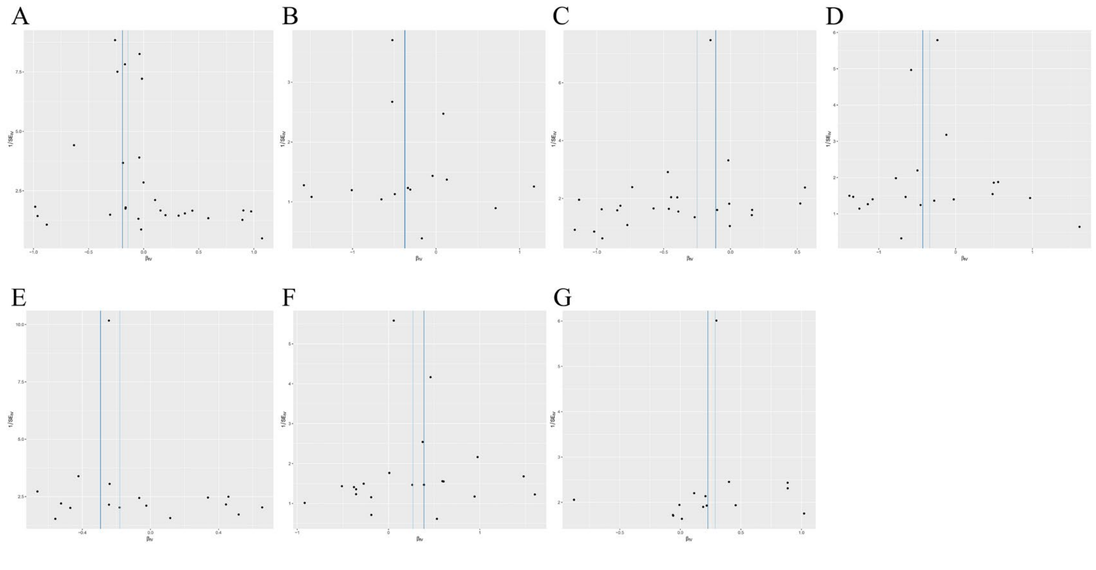

Figure S2. Funnel plot for the causal effect of immunophenotypes SNPs on the risk of GBM. (A) ebi-a-GCST90001517, (B) ebi-a-GCST90001817, (C) ebi-a-GCST90001836, (D) ebi-a-GCST90001852, (E) ebi-a-GCST90001882, (F) ebi-a-GCST90001904, (G) ebi-a-GCST90002043.

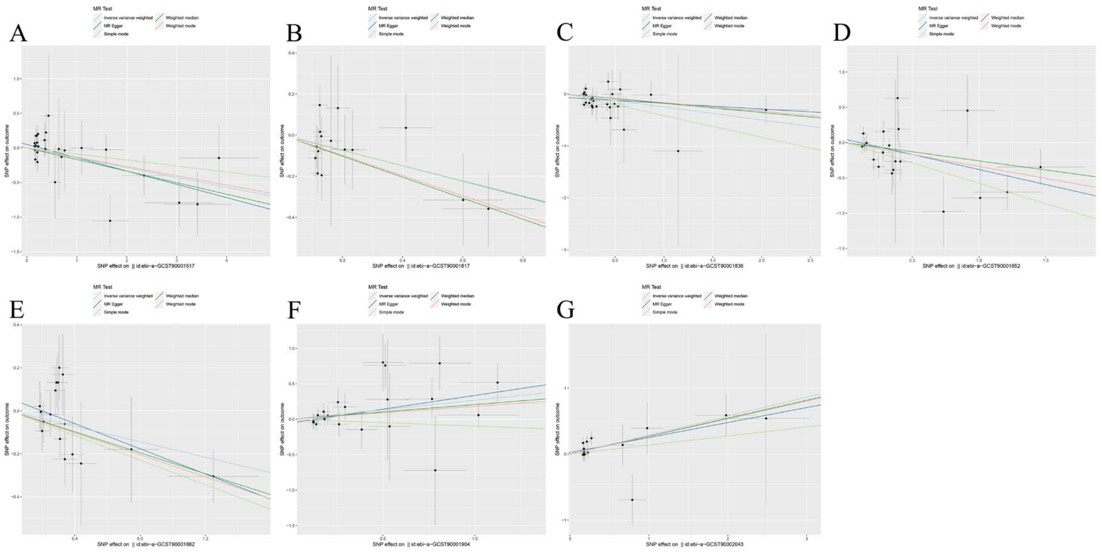

Figure S3. Scatter plot for the causal effect of immunophenotypes SNPs on the risk of GBM. (A) ebi-a-GCST90001517, (B) ebi-a-GCST90001817, (C) ebi-a-GCST90001836, (D) ebi-a-GCST90001852, (E) ebi-a-GCST90001882, (F) ebi-a-GCST90001904, (G) ebi-a-GCST90002043.

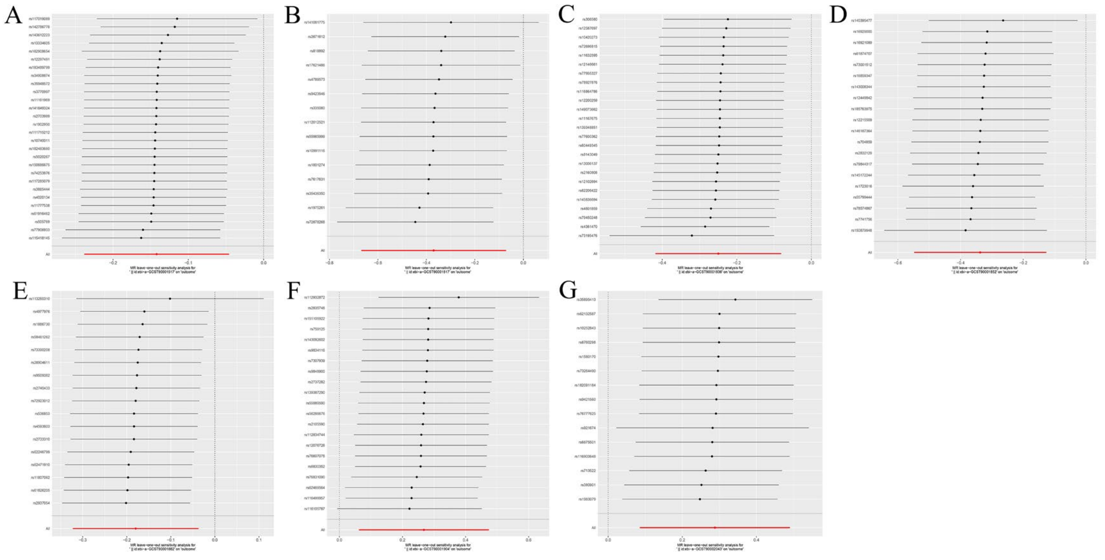

Figure S4. MR leave-one-out sensitivity analysis for the causal effect of immunophenotypes SNPs on the risk of GBM. (A) ebi-a-GCST90001517, (B) ebi-a-GCST90001817, (C) ebi-a-GCST90001836, (D) ebi-a-GCST90001852, (E) ebi-a-GCST90001882, (F) ebi-a-GCST90001904, (G) ebi-a-GCST90002043.

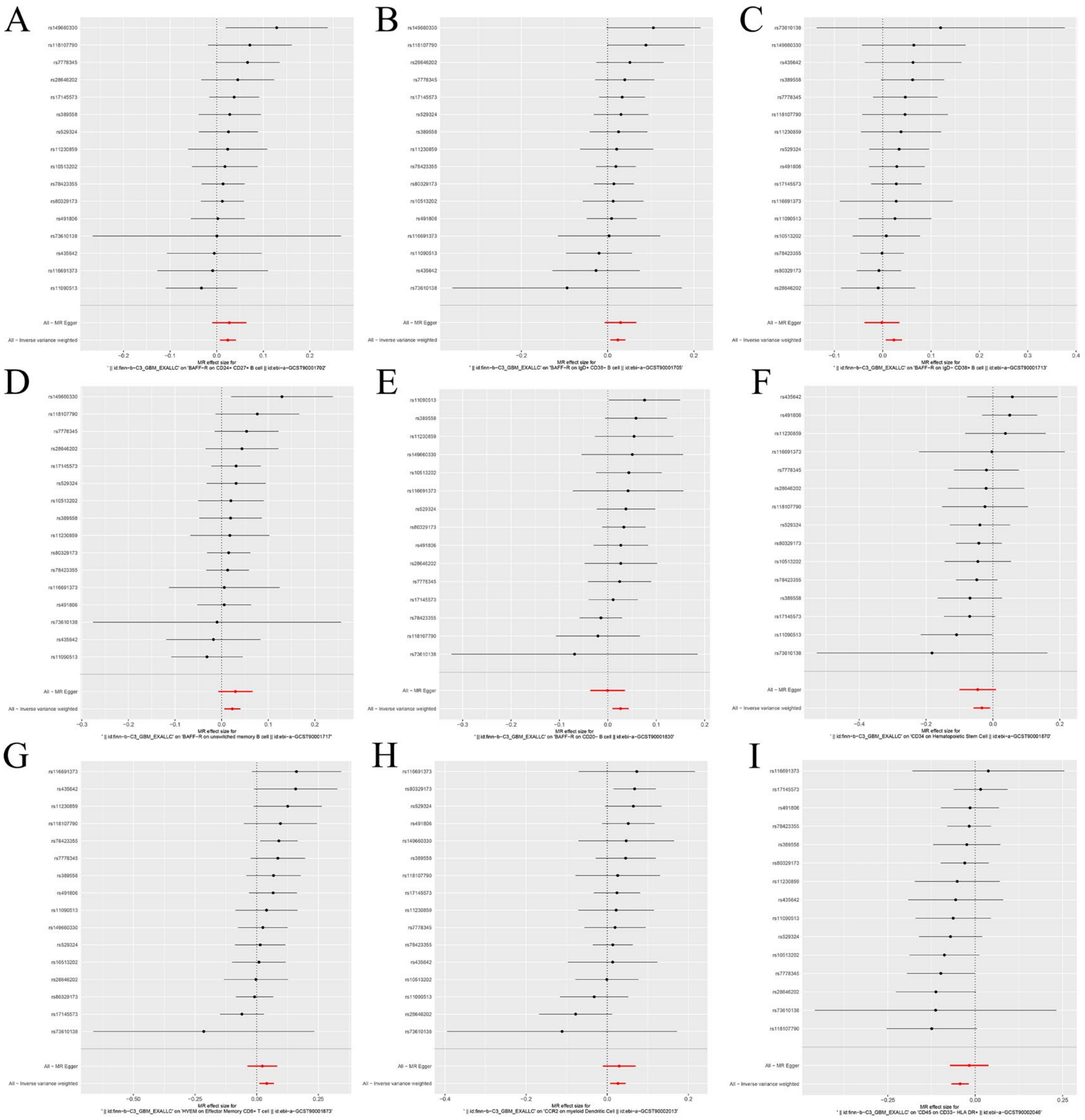

Figure S5. MR effect size for the causal effect of GBM SNPs on the risk of immunophenotypes. (A) ebi-a-GCST90001702, (B) ebi-a-GCST90001705, (C) ebi-a-GCST90001713, (D) ebi-a-GCST90001717, (E) ebi-a-GCST90001830, (F) ebi-a-GCST90001870, (G) ebi-a-GCST90001873, (H) ebi-a-GCST90002013, (I) ebi-a-GCST90002046.

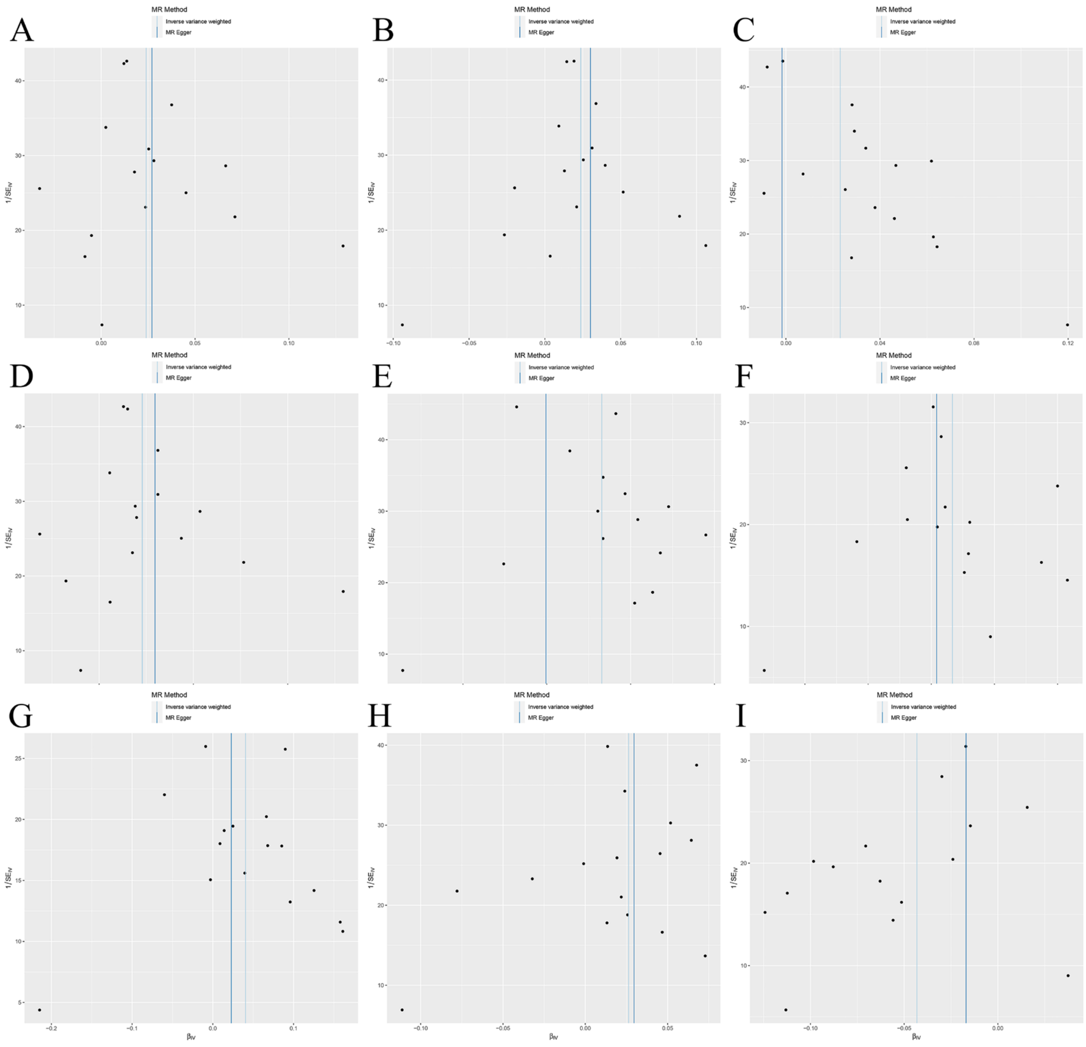

Figure S6. Funnel plot for the causal effect of GBM SNPs on the risk of immunophenotypes. (A) ebi-a-GCST90001517, (B) ebi-a-GCST90001817, (C) ebi-a-GCST90001836, (D) ebi-a-GCST90001852, (E) ebi-a-GCST90001882, (F) ebi-a-GCST90001904, (G) ebi-a-GCST90002043.

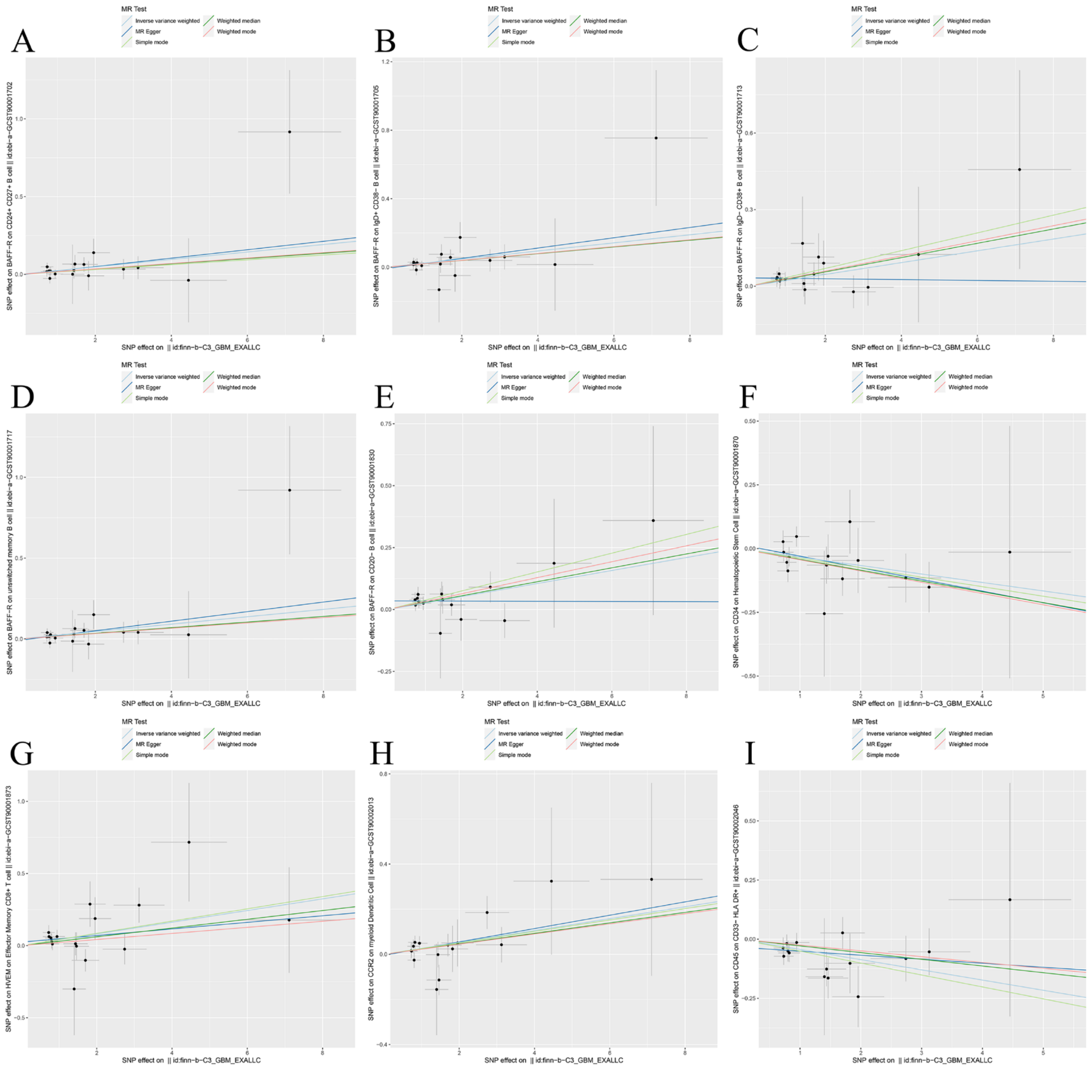

Figure S7. Scatter plot for the causal effect of GBM SNPs on the risk of immunophenotypes. (A) ebi-a-GCST90001517, (B) ebi-a-GCST90001817, (C) ebi-a-GCST90001836, (D) ebi-a-GCST90001852, (E) ebi-a-GCST90001882, (F) ebi-a-GCST90001904, (G) ebi-a-GCST90002043.

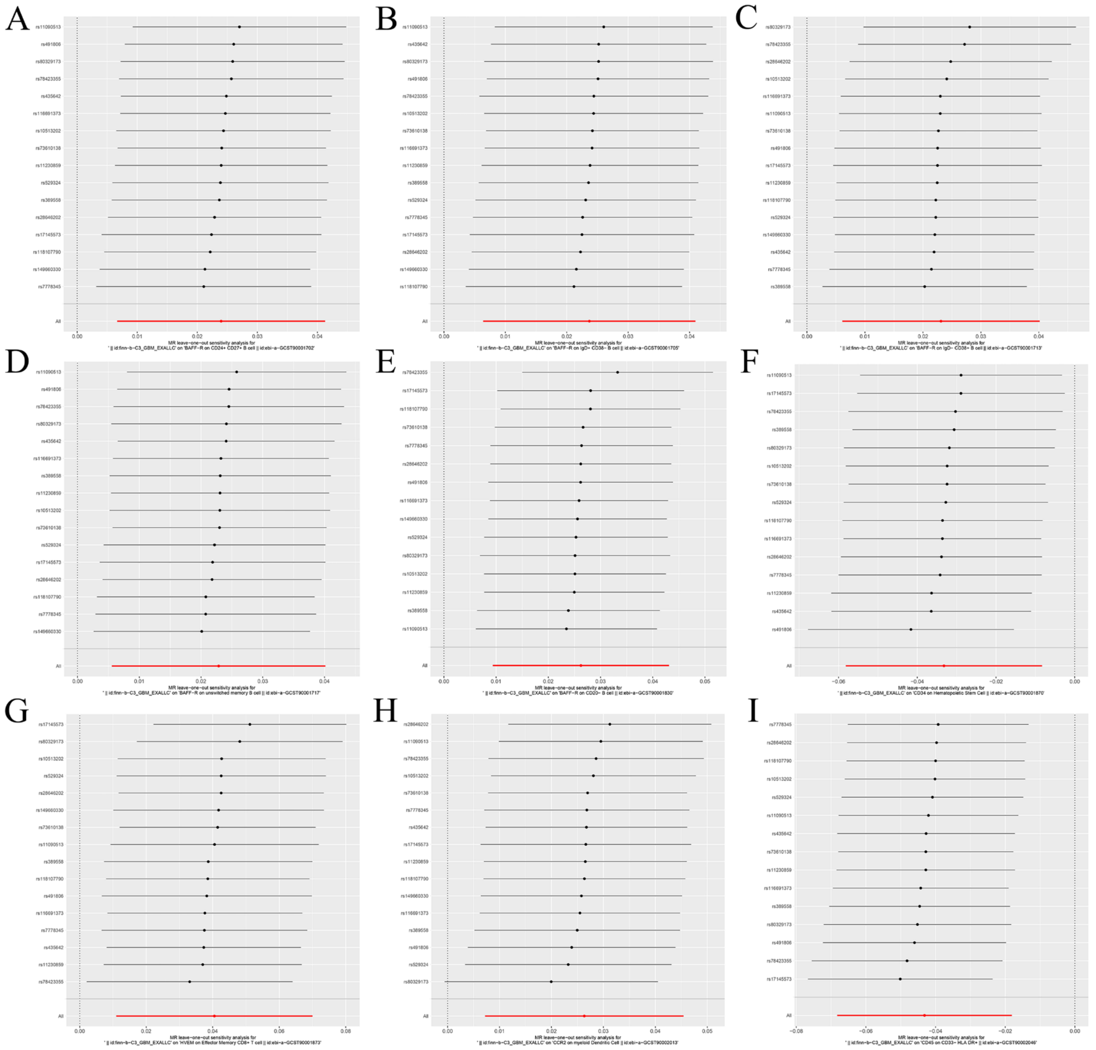

Figure S8. MR leave-one-out sensitivity analysis for the causal effect of GBM SNPs on the risk of immunophenotypes. (A) ebi-a-GCST90001517, (B) ebi-a-GCST90001817, (C) ebi-a-GCST90001836, (D) ebi-a-GCST90001852, (E) ebi-a-GCST90001882, (F) ebi-a-GCST90001904, (G) ebi-a-GCST90002043.

Table S1 GWAS data of immunophenotypes and GBM for MR

Exposure	GWAS ID	Sample size	Numbers of SNPs
CD33br HLA DR+ AC	ebi-a-GCST90001517	1858	14304991
CD38 on PB/PC	ebi-a-GCST90001817	3655	15048904
CD66b on CD66b++ myeloid cell	ebi-a-GCST90001836	1465	14033866
CD3 on CD39+ resting Treg	ebi-a-GCST90001852	2649	14779400
HVEM on CM CD8br	ebi-a-GCST90001882	1247	13730810
CD86 on CD62L+ myeloid DC	ebi-a-GCST90001904	2871	14827344
CD45 on CD33br HLA DR+ CD14dim	ebi-a-GCST90002043	1578	14127355
Brain glioblastoma	finn-b-C3_GBM_EXALLC	NA	16380303
BAFF-R on CD24+ CD27+	ebi-a-GCST90001702	3657	15048951
BAFF-R on IgD+ CD38-	ebi-a-GCST90001705	3645	15045785
BAFF-R on IgD- CD38br	ebi-a-GCST90001713	3659	15049184
BAFF-R on unsw mem	ebi-a-GCST90001717	3657	15048951
BAFF-R on CD20-	ebi-a-GCST90001830	3648	13966232
CD34 on HSC	ebi-a-GCST90001870	1634	14155797
HVEM on EM CD8br	ebi-a-GCST90001873	1247	13730810
CCR2 on myeloid DC	ebi-a-GCST90002013	2870	14824404
CD45 on CD33- HLA DR+	ebi-a-GCST90002046	1635	14155839

Table S2 Classification and attribution of immunophenotypes

Traits	Panel	Trait type
CD33br HLA DR+ AC	Myeloid cell	AC
CD38 on PB/PC	B cell	MFI
CD66b on CD66b++ myeloid cell	Myeloid cell	MFI
CD3 on CD39+ resting Treg	Treg	MFI
HVEM on CM CD8br	Maturation stages of T cell	MFI
CD86 on CD62L+ myeloid DC	cDC	MFI
CD45 on CD33br HLA DR+ CD14dim	Myeloid cell	MFI
BAFF-R on CD24+ CD27+	B cell	MFI
BAFF-R on IgD+ CD38-	B cell	MFI
BAFF-R on IgD- CD38br	B cell	MFI
BAFF-R on unsw mem	B cell	MFI
BAFF-R on CD20-	B cell	MFI
CD34 on HSC	Myeloid cell	MFI
HVEM on EM CD8br	Maturation stages of T cell	MFI
CCR2 on myeloid DC	cDC	MFI
CD45 on CD33- HLA DR+	Myeloid cell	MFI
